# Genome-wide association tests by using block information in family data

**DOI:** 10.1186/1753-6561-1-s1-s149

**Published:** 2007-12-18

**Authors:** Rui Tang, Fei Wang, Qiuying Sha, Shuanglin Zhang, Huann-Sheng Chen

**Affiliations:** 1 Department of Mathematical Sciences, Michigan Technological University, 1400 Townsend Drive, Houghton, MI 49931, USA; 2 Department of Biostatistics, University of Michigan, 1420 Washington Heights, Ann Arbor, MI 48109, USA

## Abstract

By applying an association test to analyze the data sets from Genetic Analysis Workshop 15 Problem 3, we compare power using different haplotype-block information. The results from using both of the two different coding schemes show that the test using tight blocks with limited haplotype diversity within each block is more powerful than that using evenly spaced blocks, and the latter is more powerful than that using single-marker blocks. By using carefully chosen haplotype blocks, the power of association tests may be enhanced.

## Background

Genome-wide association is a promising approach to mapping complex disease genes. Currently, either single-marker tests or haplotype-based tests are used to test association for genome-wide association studies. There is evidence that the approaches based on haplotypes are more powerful than the single-marker approaches [1]. For genome-wide association studies, a haplotype approach usually uses a sliding-window method to test one short chromosome region at a time [2]. Recent studies have suggested that linkage disequilibrium (LD) in the human genome can be partitioned into blocks with limited haplotype diversity within each block [3]. If we conduct haplotype-based tests in each haplotype block, we may gain power due to the small number of haplotypes in one haplotype block because there would be a smaller number of degrees of freedom. Furthermore, with hundreds of thousands of single-nucleotide polymorphisms (SNPs) tested for association, the *p*-values need to be adjusted for controlling type I error rates. When we test association in each block, the number of haplotype-based tests is smaller than that of single-marker tests and the correlation between haplotype-based tests is small. Thus, multiple testing would require less correction.

In this article, based on two coding schemes, we extend the general score test statistic proposed by Schaid [4] for case-parents from one child, to include multiple children. We use this extended method to test the association between a disease locus and one haplotype block. Then, by analyzing data sets in Genetic Analysis Workshop 15 (GAW15) Problem 3, we compare the power of the single-marker test and that of the haplotype-based test considering each haplotype block at a time. We also compare the power of haplotype-based tests by using different methods to find haplotype blocks. The results show that the haplotype-based approach is more powerful than the single-marker approach. When we use the haplotype-based test to test one block at a time, the haplotype diversity within the carefully chosen blocks is limited, which results in obtaining higher power than by using evenly spaced blocks.

## Methods

Consider a sample of *n *nuclear families. Suppose that there are *M *genotyped markers across the genome or in a candidate region for each sampled individual, also, that all children in the nuclear families are affected. Schaid et al. [1] proposed a general score test for association of a multi-allelic genetic marker using case-parents design. We first extend this method to include multiple diseased children in one family and deal with multi-marker haplotypes. Because each family has two diseased children in GAW15 Problem 3 data, at this point, we just consider the case with two affected children. It is straightforward to extend the approach to a general situation with more than two affected children in each family.

### General score tests for multiple children

We use *D*_1 _and *D*_2 _to represent the first and the second affected children, respectively. Let *g*_*c*1_, *g*_*c*2_, *g*_*m*_, and *g*_*f *_denote the genotypes of the first child, the second child, mother, and father, respectively. The probability of genotype of the diseased child, given the genotypes of the parents is

$$\begin{array}{l} P(g_{c1},g_{c2}|g_{m},g_{f},D_{1},D_{2})\\ =\frac{P(D_{1},D_{2}|g_{c1},g_{c2},g_{m},g_{f})P(g_{c1},g_{c2}|g_{m},g_{f})P(g_{m},g_{f})}{\sum_{g_{1}^{*}\in G}\sum_{g_{2}^{*}\in G}P(D_{1},D_{2}|g_{1}^{*},g_{2}^{*},g_{m},g_{f})P(g_{1}^{*},g_{2}^{*}|g_{m},g_{f})P(g_{m},g_{f})}\\ =\frac{P(D_{1}|g_{c1})P(D_{2}|g_{c2})}{\sum_{g_{1}^{*}\in G}\sum_{g_{2}^{*}\in G}P(D_{1}|g_{1}^{*})P(D_{2}|g_{2}^{*})}. \end{array}$$

Here, *G *is the set of the four possible genotypes the parents can produce. Choosing a baseline genotype, let *r*(*g*) be the relative risk of genotype *g *to the baseline genotype. Following Schaid et al. [1], we use log-linear model to model the relative risk, that is, *r*(*g*) = exp(*X*^*T*^*β*), with *X *representing the numerical coding of the genotype *g *(see Coding section). Then, the conditional likelihood of one family is given by

$$P(g_{c1},g_{c2}|g_{m},g_{f},D_{1},D_{2})=\frac{r(g_{c1})r(g_{c2})}{\sum_{g_{1}^{*}\in G}r(g_{1}^{*})\sum_{g_{2}^{*}\in G}r(g_{2}^{*})}.$$

If there are *n *families, denote the corresponding numerical coding of *g*_*c*1 _and *g*_*c*2 _in the *i*^th ^family as *X*_*i*1 _and *X*_*i*2_, respectively. The likelihood function can be shown as $L=\prod_{i=1}^{N}\frac{\exp(X_{i1}^{'}\beta +X_{i2}^{'}\beta )}{(\sum_{g_{i}^{*}\in G_{i}}\exp(X_{i}^{*'}\beta ){)}^{2}}$, where $X_{i}^{*}$ is the coding vector associated with a genotype $g_{i}^{*}$ and *G*_*i *_is the set of the four possible genotypes the parents of *i*^th ^family can produce. Following the general form of Rao's score test, the score test statistic *S *= *UV*^-1^*U*' has a *χ*^2 ^distribution *S *= *UV*^-1^U* ~ $\chi_{r}^{2}$, where the degrees of freedom *r *is the rank of matrix *V*, which is the information matrix of likelihood function *L *with element $V_{ij}=-E\left[\frac{\partial^{2}\ln L}{\partial \beta_{i}\partial \beta_{j}}{|}_{\beta =0}\right]$, and *U *= ∂ln*L*/∂*β*|_*β*=0_. The quantities *U *and *V *can be expressed as $U=\sum \left(X_{i1}+X_{i2}-2{\bar{X}}_{i}^{*}\right)$, $V=\sum_{i=1}^{N}2V_{i}$, with ${\bar{X}}_{i}^{*}=\frac{1}{4}\sum_{j=1}^{4}X_{ij}^{*}$, $V_{i}=\frac{1}{4}\left[\sum_{j=1}^{4}X_{ij}^{*}X_{ij}^{*'}\right]-{\bar{X}}_{i}^{*}{\bar{X}}_{i}^{*'}$, where $X_{ij}^{*}$, *j *= 1, 2, 3, 4 are the numerical coding corresponding to the four possible genotypes that the parents of the *i*^th ^family can produce.

### Coding

Suppose for one haplotype block there are *m *distinct haplotypes, denoted by *h*_1_,...,*h*_*m*_. For each person, the genotype in this block, denoted as *g*, can be a combination of any two haplotypes selected from *h*_1_,...,*h*_*m*_. Under the assumption that the phase information of the genotype is known, we use two different ways to code the genotypes.

The first coding scheme is defined as follows. Let *X *denote a *m*-dimensional indicator vector, *X *= (*x*_1_,...,*x*_*m*_). The *j*^th ^element *x*_*j*_, is the number of haplotype *h*_*j *_in the genotype *g*, so *x*_*j *_can only take three possible values – 0, 1, or 2 – when *g *has 0, 1, or 2 haplotypes *h*_*j*_, respectively. We also consider the second coding in which we test whether a specific haplotype *h*_*L *_is associated with the disease. In this case, *X *is a scalar value, taking 0, 1, or 2 when *g *has 0, 1, or 2 haplotypes *h*_*L*_, respectively. Using this coding, if there are *m *distinct haplotypes in one block, we will have *m *tests for this block. Let *p*_1_,...,*p*_*m *_denote the *p*-values of the *m *tests. In order to have an overall test between the haplotype block and the disease, we test the null hypothesis *H*_0_, where at least one haplotype is associated with the disease. The *p*-value of testing *H*_0 _is given by *p *= min{*p*_1_,...,*p*_*m*_) × *m*. Thus, using either of the two coding schemes, we have a *p*-value corresponding to each haplotype block (or a single marker).

### Select significant SNPs by controlling false-discovery rate (FDR)

Suppose we have *B *haplotype blocks. Let *P*_*i *_denote the *p*-value of the test of association between the *i*^th ^haplotype block and the disease by using the score test statistic discussed above. Denote the ordered *p*-values by *P*_(1)_,...,*P*_(*B*)_. A block is considered to be associated with the trait if its *p*-value is less than a threshold *δ*_*B*_. The threshold *δ*_*B *_is determined by controlling the FDR at level *α *[5]. The threshold *δ*_*B *_can be calculated by

$$\delta_{B}=\max\left\{P_{\left(i\right)}:P_{\left(i\right)}\leq \frac{i\alpha }{B}\right\}.$$

We choose those blocks with associated *p*-values satisfying *p *≤ *δ*_*B *_as the blocks that have a significant association with the disease.

### Haplotype blocks

One of the main objectives of this analysis is to compare the performance of the score test by using different haplotype-block information. We consider three different methods to find haplotype blocks. One method, which we call the tight block method, results in limited haplotype diversity within each block. The second method is to find evenly spaced blocks. The third method considers each single marker as a block. Many recently developed approaches can be used to find haplotype blocks with limited haplotype diversity within each block. We use a modified version of the approach developed by Zhu et al. [6] to find tight blocks. Consider two biallelic markers: marker A with alleles *A*_1 _and *A*_2 _and marker B with alleles *B*_1 _and *B*_2_. Let *p*_11 _denote the population frequency of haplotype *A*_1_*B*_1_, and $p_{A_{i}}$, $p_{B_{i}}$ denote the population frequency of allele *A*_*i *_and *B*_*i *_(*i *= 1, 2), respectively. One of the LD measures (*r*^2^), which is proportional to the statistical power of association tests, is defined by

$$r^{2}=\frac{{\left(p_{11}-p_{A_{1}}p_{B_{1}}\right)}^{2}}{p_{A_{1}}p_{B_{1}}p_{A_{2}}p_{B_{2}}}.$$

The approach in Zhu et al. [6] to find tight blocks is roughly the same as finding blocks in which all markers have a pair-wise *r *value > *r*_0_. For the purpose of the power comparison, we choose *r*_0 _= 0.2 for our analysis.

We also use the program HaploBlockFinder V0.7 [7] to find the tight blocks. The power calculations resulting from each of these two approaches are very similar. Thus, we only report the results based on tight blocks found by the approach in Zhu et al. [6].

## Results

### GAW15 data analysis

We use our proposed screening procedures to analyze the dense SNP data of chromosome 6 of the GAW15 Problem 3 simulated rheumatoid arthritis (RA) data. The data contains 100 replications total. Each includes 1500 nuclear families with two disease children and 2000 unrelated controls. In this analysis, we used only family data. For each individual, there are 17820 SNPs on chromosome 6, and the phase information for the genotype is known. From the data provided, we know that there are three disease loci – Locus DR, Locus C, and Locus D – on chromosome 6. Locus DR affects the risk of RA. Locus C increases RA risk only in woman. These two loci are in the same position. The typed SNP 3437 on chromosome 6 is in the same position where Loci DR and C are located. The rare allele of Locus D increases RA risk by five-fold. SNP 3917 is the nearest SNP to Locus D. The genetic distance between Locus D and SNP 3917 is 0.00171 cM, and the physical distance is 1565 bp. We use SNPs 3437 and 3917 as disease-associated SNPs to study the behavior of the score test by using different haplotype information.

The distributions of the blocks found by the approach in Zhu et al. [6] are given in Table 1. Most blocks have two to five markers. The average length of the haplotype block is around three markers. Thus, for the evenly spaced block, we partition the SNPs evenly with three markers in one block without using any LD measures. Comparing the two partitions, the median number of haplotypes in a block is four for a tight block partition, which is less than for evenly spaced block partitions in which the median number of haplotypes is five. The average physical length is 0.021 cM for tight block partitions and 0.026 cM for evenly spaced block partitions. The average LD in a block is 0.272 and 0.142 for tight block and evenly spaced block, respectively.

**Table 1:** The distribution of haplotype blocks using LD measure of Zhu et al. [6]

No. blocks	No. markers in each block
1	1331
2–5	2554
6–10	641
11–15	120
16–20	47
<20	20
Total	4713

The evenly spaced blocks may depend on which SNP is considered the "first" SNP. There are three possible frames of three-SNP blocks. We report the results from all three frames. Finally, we compare the two ways of partitioning with the one that does not use block information, that is, we set each marker as one block, which results in 17820 blocks in total.

### The validity of the test and power comparison

To test if the score test is valid, we consider blocks that consist of typed SNPs with id < 2000 and SNPs with id > 4500. These two regions are far away from the disease loci, and thus, they can be used to test the type I error. For each replication and each block, we calculate the *p*-values of the test. For each block scenario, the total number of tests we performed is *N *= 100 × {number of blocks}. The estimated type I error for nominal level 0.05 is given by {number of tests with *p*-value < 0.05}/*N*. From Table 2, we see that the type I error rates are very consistent with the nominal level, which indicates that the score test is valid regardless of which kind of haplotype block we use. For evenly spaced blocks, we only report the results from the frame that starts from SNP1. For the other two frames, the results are similar.

**Table 2:** Type I error rates of the tests at nominal level 0.05^a^

SNP ID	Single-marker blocks	Evenly spaced blocks	Tight blocks
The first coding			
<2000	0.051	0.063	0.052
>4000	0.044	0.047	0.039
			
The second coding			
<2000	0.042	0.078	0.050
>4000	0.036	0.060	0.050

^a^Blocks created by the method of Zhu et al. [6]

For power comparisons, we applied the test to the 100 replications and use the count of successful finding SNP 3437 or SNP 3917 in 100 replications as the power of the test to detect SNP 3437 or SNP 3917. The result is summarized in Table 3. We were able to detect SNP 3437 with power = 100% under three different block selection methods. SNP 3437 is at the same position as Locus DR and Locus C, and the association between this SNP and the disease is very strong. Therefore, the powers under three different block-selection methods and two coding schemes are all 100%. For detecting SNP 3917, the test using tight block information is more powerful than using evenly spaced blocks using either of the two coding schemes, and the latter is more powerful than using single-marker blocks. The second coding approach seems to have a better power than the first coding approach to detect SNP 3917. The reason may be that when the first coding scheme is used, the effect of a rare allele is covered by the noise of many haplotypes.

**Table 3:** The powers of the score tests using different kinds of haplotype blocks^a^

		Evenly spaced blocks^b^			
			
SNP	Single-marker blocks	SNP1	SNP2	SNP3	Tight blocks
The first coding					
3437	1.00	1.00	1.00	1.00	1.00
2917	0.27	0.37	0.28	0.24	0.43
					
The second coding					
3437	1.00	1.00	1.00	1.00	1.00
2917	0.27	0.47	0.44	0.37	0.51

^a^Blocks created by the method of Zhu et al. [6]

^b^SNP from which the block method started.

It is worth noting that for evenly spaced blocks, the results depend on which SNP is considered to be the first SNP. When SNP ID1 is considered the first SNP in the partition, SNP 3917 falls into the middle of a block, which shows the most powerful result among the three evenly spaced block formations. The power of this partition is smaller than that of the tight block partition, but is not statistically significant at level 0.05. When ID2 or ID3 are considered as the first SNP in the partition, SNP 3917 is not located in the middle of a block. They both have significantly less power than the tight block partition at level 0.05.

## Conclusion

In this paper, we first extend the score test of Schaid [4] from dealing with one affected child to the case of dealing with multiple affected children in each nuclear family. Applying this test to the dense SNP data in GAW15 Problem 3, we compared the power of the test by using different haplotype block information. The conclusion we reach is that the test using tight block with limited haplotype diversity within each block is more powerful than that using evenly spaced blocks, and the latter is more powerful than that using single-marker blocks. The reason may be that, when using tight blocks, there is limited diversity within each block, and thus the degrees of the freedom of the test is small, which may in turn increase the power of the test.

One thing we need to mention is that for the multi-marker blocks (tight block and evenly spaced block) we assume that the phase information is known. Further investigation is needed to evaluate the performance of the test using multi-marker blocks when the phase information is unknown.

## Competing interests

The author(s) declare that they have no competing interests.
